# Proposal of a New Standardized Freeze-Thawing Technical Protocol for Leucocyte-Poor Platelet-Rich Plasma Preparation and Cryopreservation

**DOI:** 10.7759/cureus.8997

**Published:** 2020-07-04

**Authors:** André Caiado, Guilherme Ferreira-Dos-Santos, Sérgio Gonçalves, Luís Horta, Pedro Soares Branco

**Affiliations:** 1 Immunohemotherapy, Central Lisbon University Hospital Center, Lisbon, PRT; 2 Physical Medicine and Rehabilitation, Central Lisbon University Hospital Center, Lisbon, PRT; 3 Physical Medicine and Rehabilitation, Nova Medical School, Nova University of Lisbon, Lisbon, PRT; 4 Orthopedic Surgery, Central Lisbon University Hospital Center, Lisbon, PRT

**Keywords:** freeze-thawing, pain management, platelet activation, platelet-rich plasma, regenerative medicine, regenerative medicine therapies, technical report

## Abstract

A human platelet-rich plasma (PRP) concentrate can be defined as a preparation of autologous human plasma with increased platelet concentration produced by centrifugation of a larger volume of a patient’s own blood. Platelets contain a plethora of growth factors in their α-granules that are concentrated through the centrifugation process in order to then be injected in supraphysiologic amounts to an injury site with the final aim of augmenting the natural healing process. Preparations of PRP concentrates can be further classified as leucocyte-rich (LR-PRP), defined as having a leucocyte concentration above baseline, and leucocyte-poor (LP-PRP), defined as having a leucocyte concentration below baseline. Although many preclinical and clinical trials have shown the ability of leucocyte-poor PRP concentrates to significantly improve symptomatic mild to moderate hip and/or knee osteoarthritis, to date there is no consensus on the optimal way of obtaining PRP preparations, specifically with respect to the concentration of blood components. In this technical report, we describe a new standardized freeze-thawing technique for leucocyte-poor PRP preparation and cryopreservation, which has been shown to be superior to currently available techniques based solely on centrifugation. By describing this technical protocol, which we have been using on a daily basis in the setting of a Regenerative Medicine Outpatient Clinic in a European tertiary university hospital center, we aim to contribute to a future consensus on the optimal way of obtaining and preserving leucocyte-poor PRP concentrates.

## Introduction

A human platelet-rich plasma (PRP) concentrate can be defined as a preparation of autologous human plasma with increased platelet concentration produced by centrifugation of a larger volume of a patient’s own blood [1].

Platelets contain a plethora of growth factors in their α-granules: epidermal growth factor (EGF), basic fibroblast growth factor (bFGF), hepatocyte growth factor (HGF), platelet-derived growth factor AB (PDGF-AB), transforming growth factor β1 (TGF-β1), and vascular endothelial growth factor (VEGF). When preparing a PRP syringe, platelets are concentrated through the centrifugation process in order to then be injected in supraphysiologic amounts to an injury site with the final aim of augmenting the natural healing process [1,2].

Preparations of PRP concentrates can be further classified as leucocyte-rich (LR-PRP) preparations, defined as having a leucocyte concentration above baseline, and leucocyte-poor (LP-PRP) preparations, defined as having a leucocyte concentration below baseline [1].

Although preclinical and clinical trials have shown the ability of LP-PRP concentrates to significantly improve symptomatic mild to moderate hip and/or knee osteoarthritis, to date there is no consensus on the optimal way of obtaining PRP preparations, specifically with respect to concentration of blood components. As such, variation exists in PRP collection protocols and preparation characteristics depending on the system utilized [1,3].

We describe a standardized freeze-thawing technique for LP-PRP preparation and cryopreservation, which has been shown to be superior to currently available techniques based solely on centrifugation (with regards to the effectiveness of platelet activation and subsequent release of α-granule growth factors). By describing our technical protocol, which we have been using on a daily basis in the setting of a Regenerative Medicine Outpatient Clinic in a European tertiary university hospital center, we hope to contribute to a future consensus on the optimal way of obtaining and preserving LP-PRP concentrates.

This report was first presented at the sixth Annual Meeting and Interventional Pain Workshop of the World Academy of Pain Medicine United (WAPMU - previously, the World Academy of Pain Medicine Ultrasonography), in Miami, USA, where it was awarded the first Annual Gofeld Academic Scholarship Award by the Board of Education of the WAPMU [4].

## Technical report

A) Standard technique

Candidates are submitted to a pre-collection clinical evaluation and blood testing for hemoglobin concentration, leucocyte and platelet count, human immunodeficiency virus (HIV), hepatitis B virus (HBV), hepatitis C virus (HCV) and Treponema pallidum screening, in accordance with the Council of Europe's regulations for autologous blood donation [5].

Approved patients undergo peripheral venous collection of 450 milliliters of whole blood using a four-bag collection system with an in-line platelet leucocyte-depletion filter. Whole blood is then processed by refrigerated centrifugation in a Heraeus™ Cryofuge™ 6000i (Heraeus Instruments, Osterode, Germany) (centrifuge parameters should be individually determined for the equipment in use). A soft spin (relative centrifugal force of 1328 x g, in our centrifuge: 2000 rpm for 10 minutes at 20±2 ºC) separates PRP from packed red blood cells which are discarded. A subsequent heavy spin (relative centrifugal force of 4303 x g, in our centrifuge: 3600 rpm for 15 minutes at 20±2 ºC) separates PRP from platelet-poor plasma (PPP) which is discarded. Using sterile connections, the resulting LP-PRP preparation of approximately 60 mL is then divided into four different syringes, each containing 6 milliliters of the concentrate. The remaining content of the bag is submitted to quality control in accordance with mandatory European regulations for platelet concentrates. All products thus obtained are tested for residual leucocytes, platelet count and bacteriologic cell culture (for aerobes and anaerobes). The methods here described for component preparation from whole blood are in accordance with the methods in use in our centre for whole blood donations, therefore complying with internationally accepted standards [5,6].

**Figure 1:**
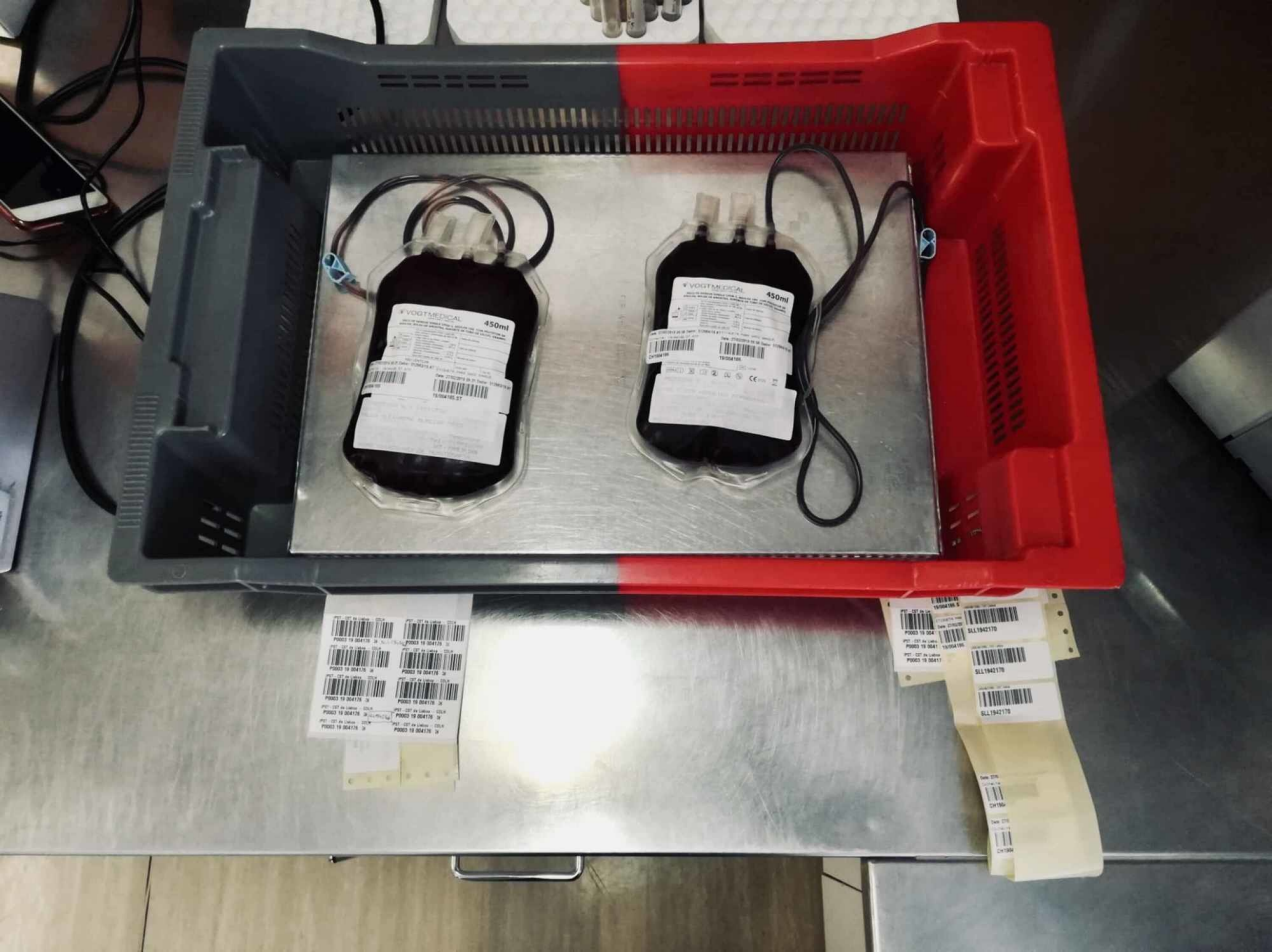
Step A of the standardized freeze-thawing technical protocol for leucocyte-poor platelet-rich plasma (LP-PRP) preparation and cryopreservation. Whole blood unit resting on a butane-1,4-diol cooling plate after venous collection.

**Figure 2:**
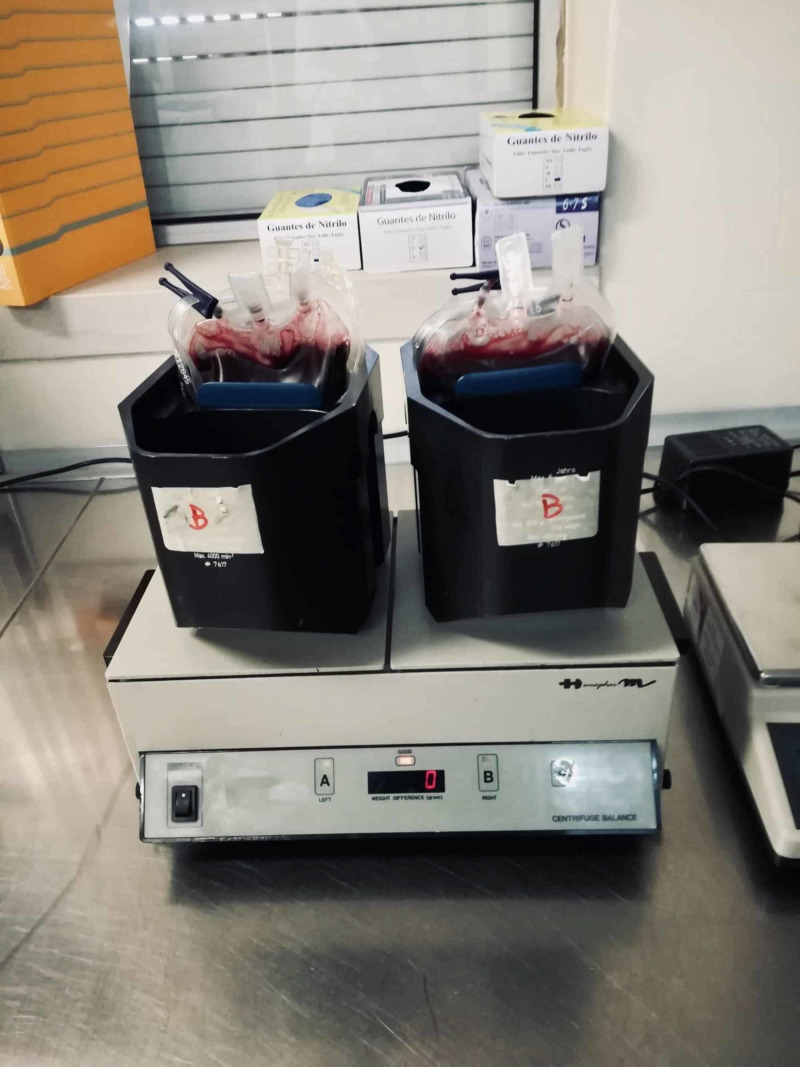
Step B of the standardized freeze-thawing technical protocol for leucocyte-poor platelet-rich plasma (LP-PRP) preparation and cryopreservation. Weighing and first centrifugation (soft spin) of the whole blood unit.

**Figure 3:**
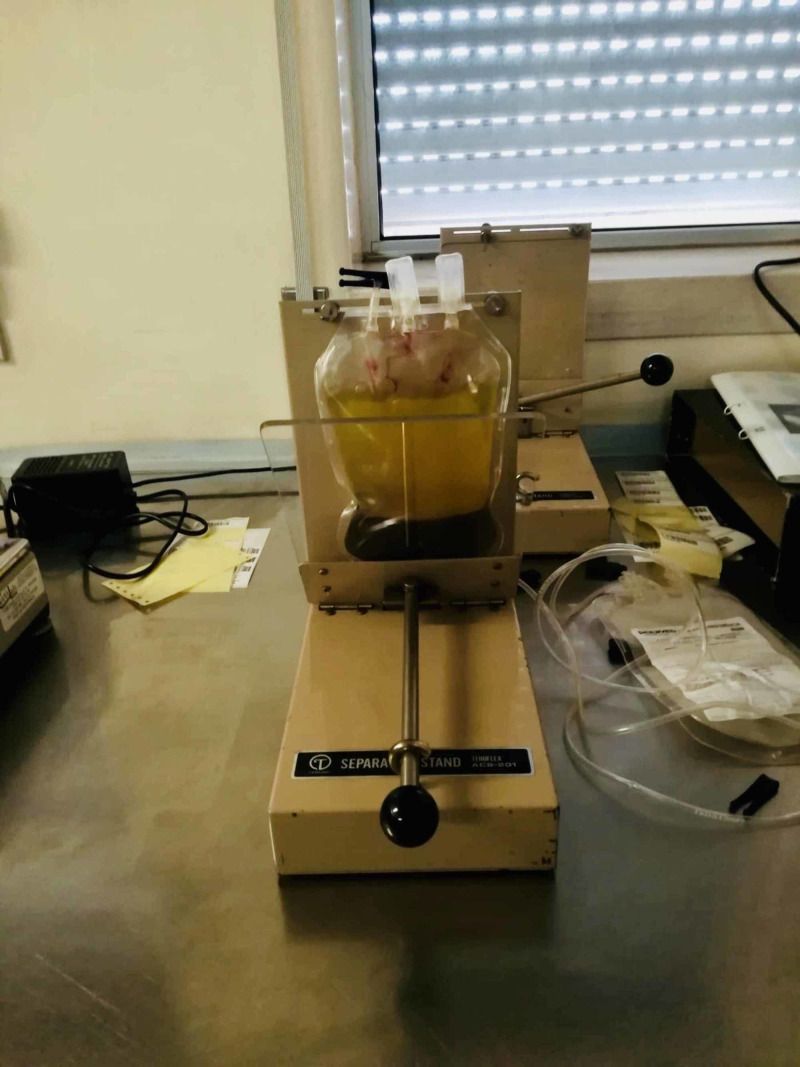
Step C of the standardized freeze-thawing technical protocol for leucocyte-poor platelet-rich plasma (LP-PRP) preparation and cryopreservation. First separation of platelet-rich plasma (PRP) from packed red blood cells (discarded) after the first centrifugation (soft spin).

**Figure 4:**
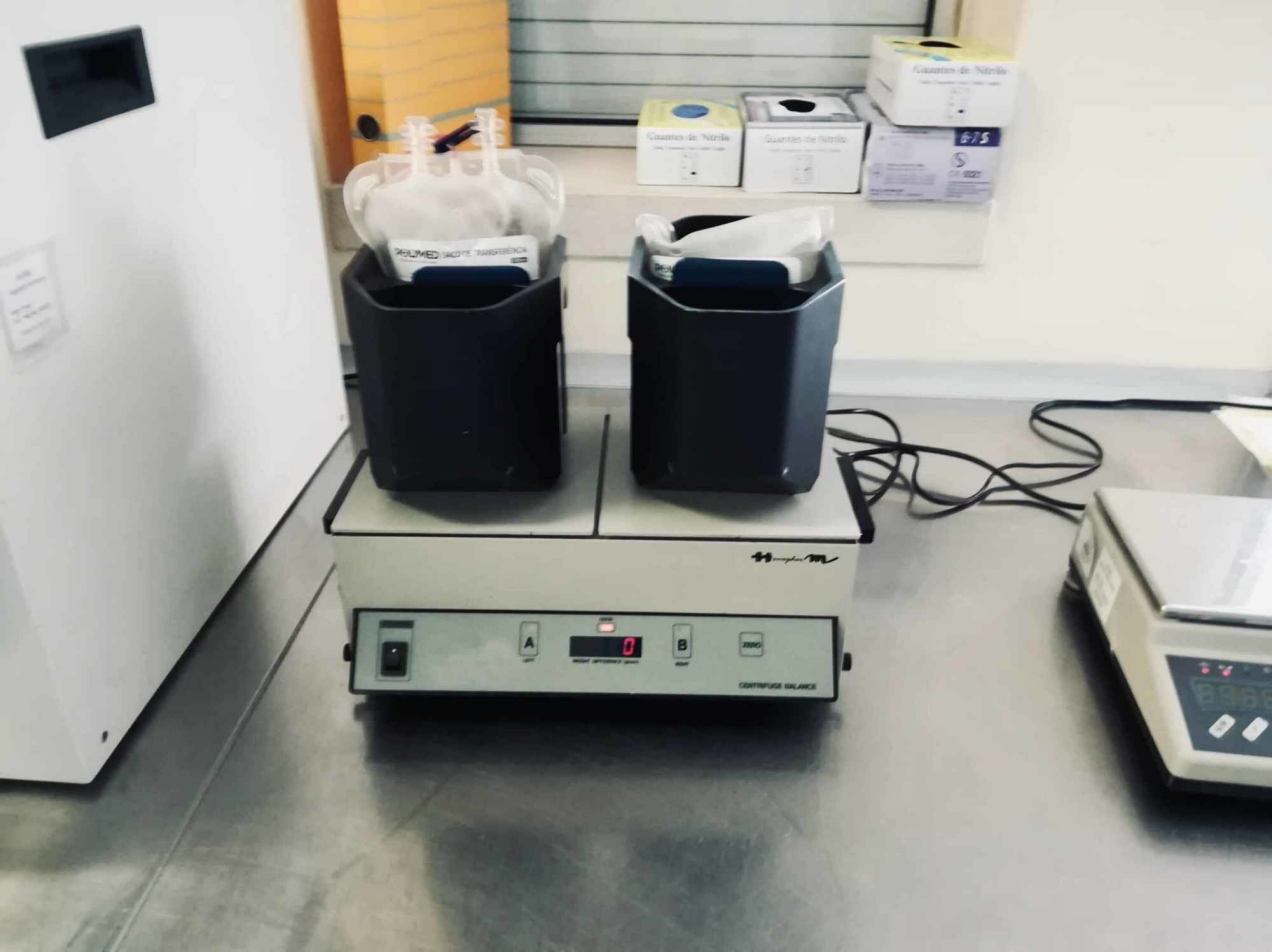
Step D of the standardized freeze-thawing technical protocol for leucocyte-poor platelet-rich plasma (LP-PRP) preparation and cryopreservation. Weighing and second centrifugation (heavy spin), which separates platelet-rich plasma (PRP) from platelet-poor plasma (PPP) (discarded).

**Figure 5:**
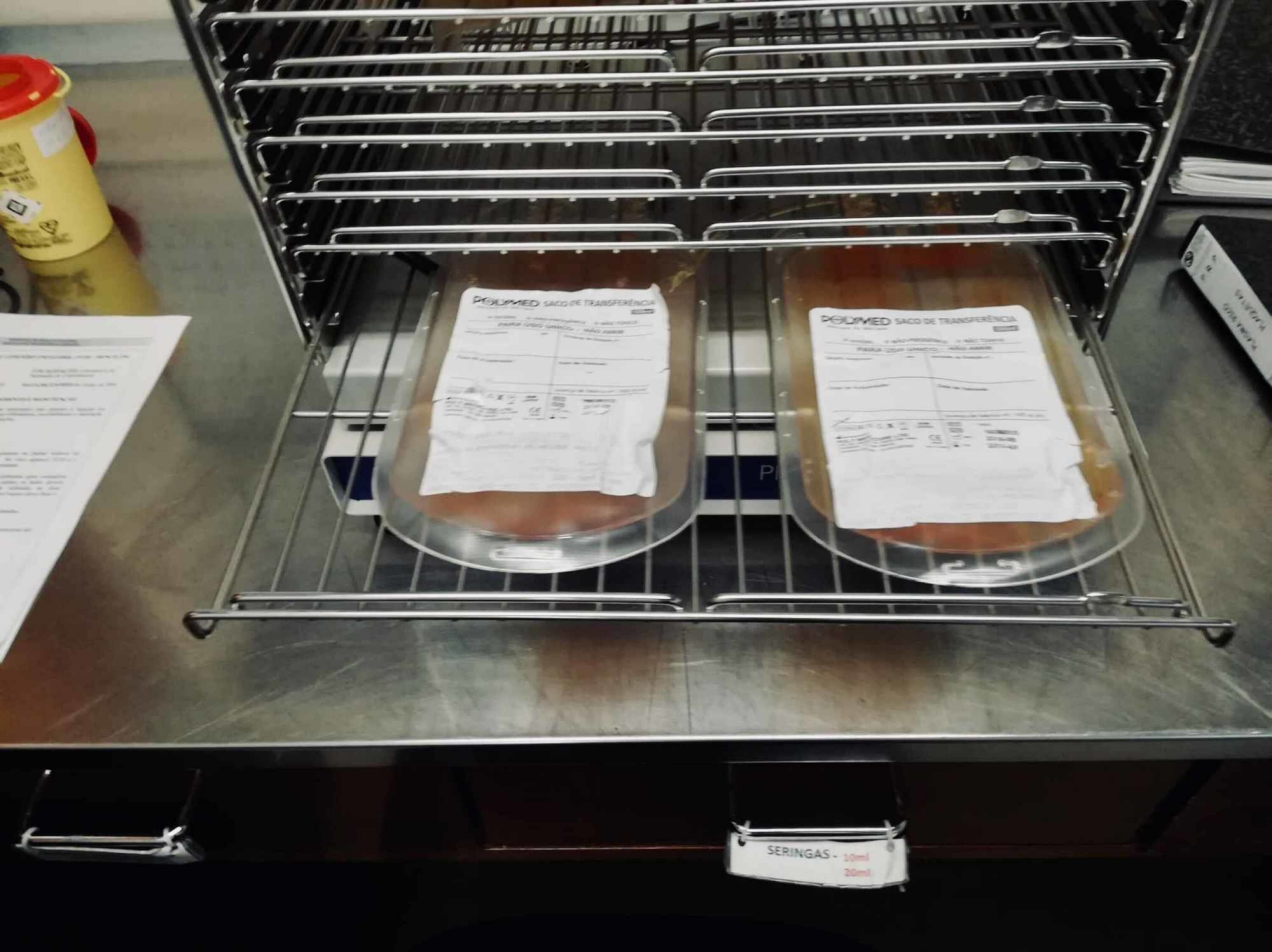
Step E of the standardized freeze-thawing technical protocol for leucocyte-poor platelet-rich plasma (LP-PRP) preparation and cryopreservation. LP-PRP concentrate after the second centrifugation and separation.

**Figure 6:**
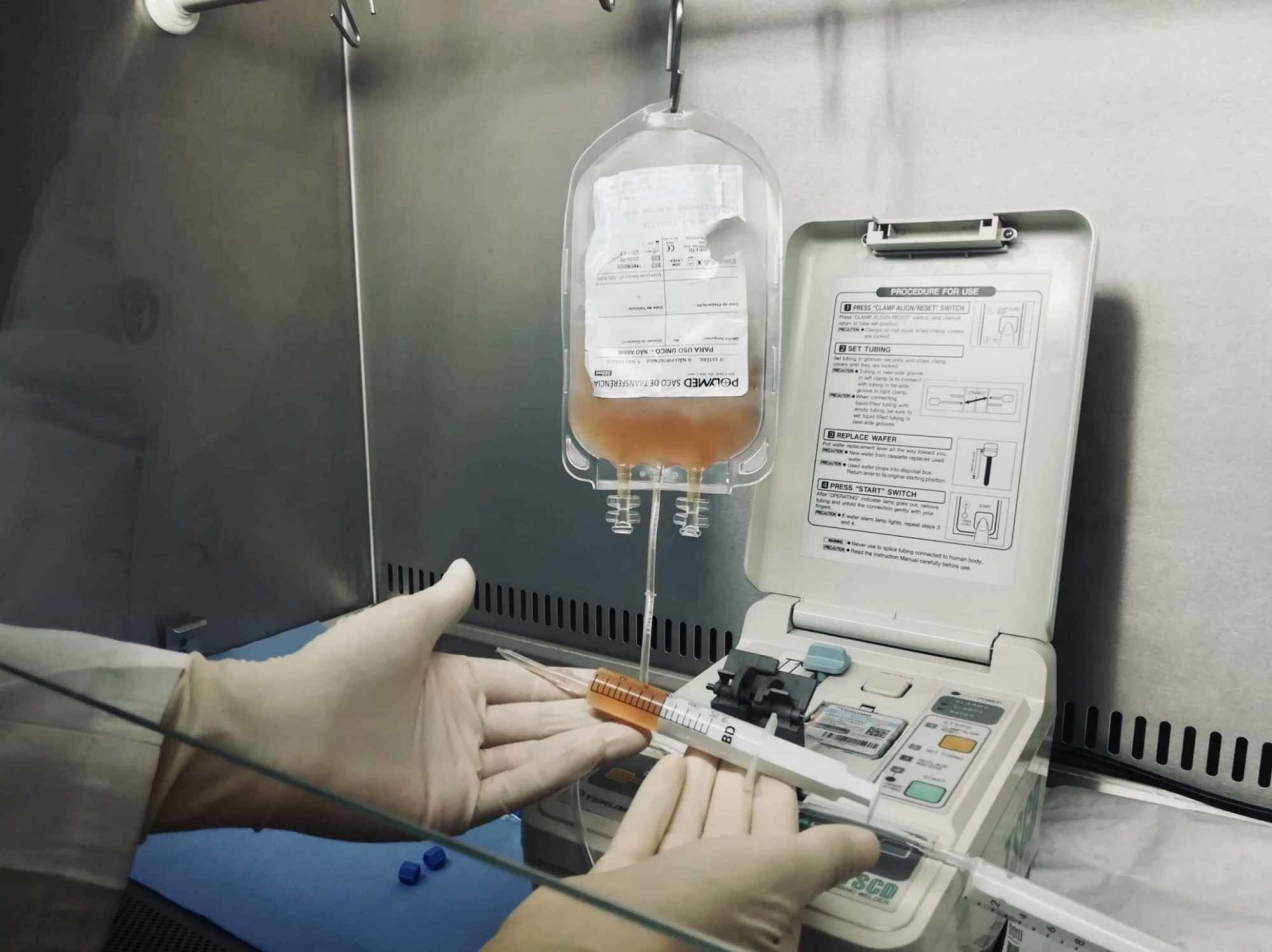
Step F of the standardized freeze-thawing technical protocol for leucocyte-poor platelet-rich plasma (LP-PRP) preparation and cryopreservation. LP-PRP concentrate in an individual syringe, ready to be labeled and submitted to three repeated cycles of freeze-thawing at temperatures between -30 and -20 degrees Celsius.

The final four syringes containing LP-PRP are then submitted to three repeated cycles of freeze-thawing at temperatures between -30 and -20 degrees Celsius. Finally, after three repeated cycles of freeze-thawing, the LP-PRP syringes are cryopreserved at temperature below -20 degrees Celsius in a plasma refrigerator and can remain stored this way for at least one year (Figures 1-6).

**Figure 7:**
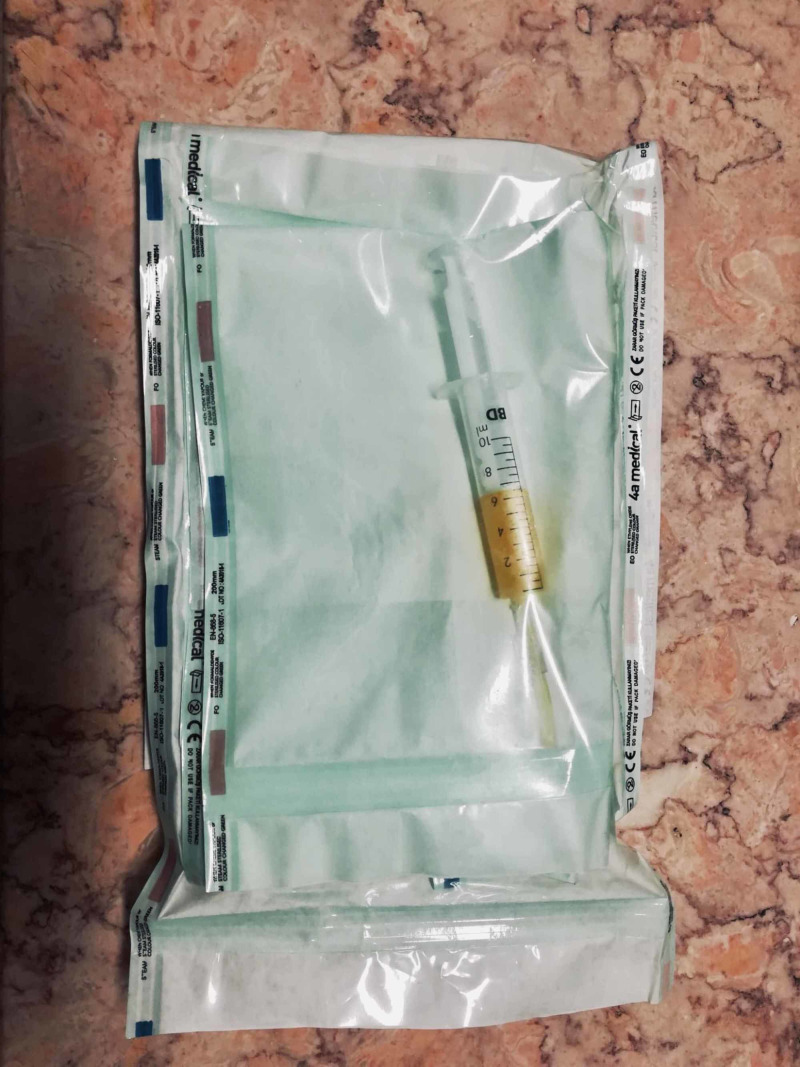
Step G of the standardized freeze-thawing technical protocol for leucocyte-poor platelet-rich plasma (LP-PRP) preparation and cryopreservation. End product (cryopreserved individual syringe with LP-PRP concentrate) ready for application after being submitted to three repeated cycles of freeze-thawing at temperatures between -30 and -20 degrees Celsius.

On the day of administration, the LP-PRP syringe is thawed in a lukewarm water bath for 8 to 12 minutes or using a fresh frozen plasma thawing device and applied within the first 60 minutes (Figure 7).

The whole technique, from collection to injection of the LP-PRP, is performed in closed system, therefore minimizing the possibility of bacterial contamination and/or growth. Identification of the autologous blood products and storing of all clinical data and cryopreserved syringes containing LP-PRP is performed in accordance with European regulations (Video 1) [5].

**Video 1:** Standardized freeze-thawing technical protocol for LP-PRP preparation and cryopreservation. This video describes the technical protocol conceived by our clinical team at our Regenerative Medicine Outpatient Clinic for leucocyte-poor platelet-rich plasma (LP-PRP) preparation and cryopreservation.

B) One-day timeframe technique

In countries where autologous blood derivatives have to be collected and administered in the same day (thus excluding the possibility of long-term cryopreservation), it is possible to implement a variation of this technical protocol, so that this entire procedure from whole blood collection to LP-PRP injection can be performed in a one-day timeframe.

To perform this technique in a one-day timeframe, the following modifications from standard technique are needed: A) considering a single LP-PRP injection scenario (5-6 mL), a venous blood collection of 80 mL suffices; B) when performed by an experienced laboratory technician, it takes approximately four hours to prepare a whole blood sample for cryopreservation following all the steps of the previously described standard technique (multiple samples from different patients can be prepared at the same time, improving labor efficiency in the laboratory); C) freezing a LP-PRP concentrate syringe to temperatures below -20 degrees Celsius in a general hospital blood bank freezer takes between 50 and 60 min. It then takes between 8 and 12min to thaw a LP-PRP concentrate syringe in a lukewarm water bath.

Taking the previous modifications from standard technique into account, and considering a whole blood collection performed at 08:00 am, it is possible to have the final LP-PRP syringe ready for application at 03:30-04:00 pm of the same day, having performed three repeated cycles of freeze-thawing.

## Discussion

To date, several different physiological/biochemical and mechanical methods of in vitro platelet activation have been tested (coagulation with CaCl2, activation with adenosine diphospate (ADP), fibrillar collagen type I, thrombin, thrombin receptor activating peptide, or zeolite). Of all the methods studied, techniques that involve freeze-thawing of platelet suspensions have been proven to be the most effective procedures [7,8].

Freeze-thawing protocols induce platelet activation by hypo-osmotic shock and repeated freeze-thawing cycles. These methods have been established as being superior to techniques based solely on centrifugation (with or without intermediate careful washing steps), with regards to the release of α-granule growth factors and P-selectin. Platelet concentrates obtained by freeze-thawing techniques were shown to contain higher concentrations of the α-granule derived growth factors EGF, bFGF, HGF, PDGF-AB, TGFβ1, and VEGF. This was first demonstrated in 1985 by Baraño and Hammond, who proved that maximization of platelet activation was achieved when submitting platelet suspensions to three repeated cycles of freeze-thawing at temperatures lower than -20 degrees Celsius [3,7].

More recently, Rauch et al. (2011) demonstrated that freeze-thawing protocols were the only methods of in vitro platelet activation that achieved maximal release of α-granule growth factors, while procedures based solely on centrifugation techniques (even with careful washing steps) did not achieve the same results [8]. In their work, the authors first utilized scanning electron microscopy (SEM) and demonstrated that human thrombocytes submitted to freeze-thawing methods showed morphological changes that indicated platelet activation, altering their shape from spheroid-discoid to pseudopodia-like. Then, by utilizing P-selectin as a marker for α-granule growth factor release, the authors performed an immunoblot comparing the presence/absence of P-selectin bands in three different samples: A) resting platelets; B) a platelet suspension submitted to centrifugation and careful washing; C) a platelet suspension submitted to centrifugation and three cycles of repeated freeze-thawing at -20 degrees Celsius. The immunoblot showed that only the platelet suspension submitted to three cycles of freeze-thawing demonstrated clear bands of P-selectin, indicating that only in this sample was α-granule degranulation and subsequent growth factor release maximal. Additionally, in the same set of experiments the authors also demonstrated that when stored at temperatures below -20 degrees Celsius, platelet concentrates maintained their stability for long periods of time [8].

This technical protocol utilizes equipment and methods available and validated for use in any hospital blood bank. Therefore, it precludes the need for commercial kits. Moreover, for each whole blood collection performed, this technique allows for a minimum preparation of four LP-PRP concentrates, which can be cryopreserved for 12 months after collection. From a cost-benefit standpoint, the preparation of the four LP-PRP syringes (obtained from one whole blood collection) costs our center 110€ (excluding labor costs), while the prices of commercial PRP kits (highly variable between countries and brands) start at around 50-60€ in Southern Europe. This means that, while the first LP-PRP injection is more expensive when utilizing our protocol, when compared to the use of commercial kits, subsequent injections in the same patient become considerably cheaper.

Another important aspect to take into consideration is that there are several benefits to the utilization of this technical protocol other than the maximization of α-granule degranulation and subsequent growth factor release: A) Firstly, by performing a platelet count of the whole blood sample and the final LP-PRP concentrate, this technique allows for a precise comparison of the degree of platelet concentration in the final LP-PRP concentrate syringe versus the patient’s baseline. This makes it easier to design accurate clinical trials that aim to compare the effectiveness of LP-PRP concentrates with different platelet concentrations; B) Secondly, preparation and long-term cryopreservation of multiple LP-PRP syringes prepared from the same whole blood collection in a blood bank ensures that the patient can be submitted to more than one injection in the period of 12 months without the need for subsequent collections of venous blood samples, and ensures that all the injections are performed with exactly the same concentration of platelets collected from the same sample, making it easier to design accurate clinical trials that aim to compare the effectiveness of single versus multiple LP-PRP concentrate injections (in countries/states where autologous blood derivatives can be collected and cryopreserved for future utilization); C) Finally, by performing this entire technical protocol in a fully closed-system in a certified hospital blood bank, biological sample contamination risk is minimized throughout the process, therefore minimizing the risk of post-injection complications for the patient.

This report was presented as a poster [4]. (Poster: Ferreira-Dos-Santos G, Caiado A, Gonçalves SR, Horta L, Soares Branco P. Standardization and 6-Month Preliminary Clinical Data on a New Freeze-Thawing Technical Protocol for Leucocyte-Poor Platelet-Rich Plasma Preparation and Cryopreservation for Utilization in Pain Medicine. WAPMU 6th Annual Meeting and Workshops; February 2020)

## Conclusions

The technical protocol hereby described has been successfully utilized in the preparation of over 100 LP-PRP injections performed in more than 70 patients, in the setting of our Regenerative Medicine Outpatient Clinic, since January 2019. The LP-PRP concentrates thus obtained have been applied in the treatment of patients presenting with chronic mild to moderate hip and/or knee osteoarthritis. Having described the technical protocol for this new freeze-thawing standardized method for LP-PRP preparation and cryopreservation in a step-by-step guideline, we hope that this approach can now be replicated in other healthcare institutions. This could lead to a wider acceptance and future development of new technical approaches to LP-PRP preparation and cryopreservation, possibly contributing to a future consensus on an optimal way of obtaining LP-PRP concentrates.
